# Single and combined strategies for mesenchymal stem cell exosomes alleviate liver fibrosis: a systematic review and meta-analysis of preclinical animal models

**DOI:** 10.3389/fphar.2024.1432683

**Published:** 2024-07-31

**Authors:** Xiaolei Zhou, Yan Xu, Xuesong Wang, Wenming Lu, Xingkun Tang, Yu Jin, Junsong Ye

**Affiliations:** ^1^ Subcenter for Stem Cell Clinical Translation, First Affiliated Hospital of Gannan Medical University, Ganzhou, Jiangxi, China; ^2^ School of Rehabilitation Medicine, Gannan Medical University, Ganzhou, Jiangxi, China; ^3^ Ganzhou Key Laboratory of Stem Cell and Regenerative Medicine, Ganzhou, Jiangxi, China; ^4^ Jiangxi Provincal Key Laboratory of Tissue Engineering (2024SSY06291), Gannan Medical University, Ganzhou, Jiangxi, China; ^5^ Key Laboratory of Prevention and Treatment of Cardiovascular and Cerebrovascular Diseases, Ministry of Education, Gannan Medical University, Ganzhou, Jiangxi, China

**Keywords:** mesenchymal stem cell exosomes, liver fibrosis, efficacy, combination drugs, meta-analysis

## Abstract

**Background:** The efficacy of mesenchymal stem cells (MSCs) in treating liver fibrosis has been supported by various clinical studies. However, stem cell transplantation is limited in clinical application due to its low survival rate, low liver implantation rate, and possible carcinogenicity. Recently, there has been increasing interest in the use of MSC-exos due to their widespread availability, low immunogenicity, and non-carcinogenic properties. Numerous studies have demonstrated the potential of MSC-exos in treating liver fibrosis and preventing progression to end-stage liver disease.

**Objective:** This study aimed to systematically investigate the efficacy of MSC-exos single administration in the treatment of hepatic fibrosis and the combined advantages of MSC-exos in combination with drug therapy (MSC-exos-drugs).

**Methods:** Data sources included PubMed, Web of Science, Embase, and the Cochrane Library, which were built up to January 2024. The population, intervention, comparison, outcomes, and study design (PICOS) principle was used to screen the literature, and the quality of the literature was evaluated to assess the risk of bias. Finally, the data from each study’s outcome indicators were extracted for a combined analysis.

**Results:** After screening, a total of 18 papers (19 studies) were included, of which 12 involved MSC-exos single administration for the treatment of liver fibrosis and 6 involved MSC-exos-drugs for the treatment of liver fibrosis. Pooled analysis revealed that MSC-exos significantly improved liver function, promoted the repair of damaged liver tissue, and slowed the progression of hepatic fibrosis and that MSC-exos-drugs were more efficacious than MSC-exos single administration. Subgroup analyses revealed that the use of AD-MSC-exos resulted in more consistent and significant efficacy when MSC-exos was used to treat hepatic fibrosis. For MSC-exos-drugs, a more stable end result is obtained by kit extraction. Similarly, infusion through the abdominal cavity is more effective.

**Conclusion:** The results suggest that MSC-exos can effectively treat liver fibrosis and that MSC-exos-drugs are more effective than MSC-exos single administration. Although the results of the subgroup analyses provide recommendations for clinical treatment, a large number of high-quality experimental validations are still needed.

**Systematic Review Registration:** CRD42024516199.

## 1 Introduction

Liver fibrosis poses a significant obstacle in the management of liver disorders, with hepatic stellate cell (HSC) activation being a key feature. HSCs are active between hepatocytes and liver sinusoidal endothelial cells, and they have the ability to store vitamins and regulate blood flow to the hepatic sinusoid at rest (Saeed et al., 2021). When liver injury occurs, on the one hand, neighboring cells release inflammatory factors in a paracrine manner to activate HSCs, and on the other hand, the inflammatory environment can stimulate hepatic macrophages to secrete IL-1β and IL-6 to further activate HSCs. Immediately thereafter, activated hepatic stellate cells (aHSCs) are able to maintain their activated state both by secreting transforming growth factor β (TGF-β) and transforming into myofibroblasts that secrete collagen type I (COL-I), collagen type III (COL-III), and α-smooth muscle actin (α-SMA) (Lee et al., 2021). Moreover, aHSCs can also increase the secretion of tissue inhibitor of metalloproteinases (TIMPs) to promote collagen deposition. Eventually, with the proliferation and migration of HSCs and the continuous accumulation of the extracellular matrix, hepatic fibrosis gradually forms. Given the intricate nature of liver fibrosis progression, clinical treatment faces significant challenges, underscoring the critical need for further research and development of improved therapeutic strategies for this condition.

Mesenchymal stem cells (MSCs), a class of pluripotent stem cells with proliferative and differentiation potential, are widely distributed in the bone marrow, adipose tissue, and umbilical cord tissue. Numerous studies have shown that MSCs can migrate to the site of liver injury to reverse hepatic fibrosis through immunomodulation, hepatogenic differentiation, and paracrine mechanisms. The therapeutic mechanisms include 1) the secretion of hepatocyte growth factor (HGF), tumor necrosis factor α (TNF-α), and other cytokines to stimulate the proliferation of hepatocytes and enhance their functions; 2) the inhibition of immune cell proliferation through cell-to-cell contact or the secretion of factors; 3) MSCs improve liver function by inhibiting the proliferation of aHSCs and stimulating their apoptosis; 4) MSCs can be directed to differentiate into liver-like cells to replace apoptotic hepatocytes (Guo et al., 2016). Despite the promising efficacy of MSCs in liver disease treatment, concerns about their potential carcinogenicity and cancer-promoting properties have limited their clinical use.

Mesenchymal stem cell exosome (MSC-exos) therapy offers a promising solution to the challenges associated with liver fibrosis treatment. MSC-exos provide three key therapeutic advantages over MSCs. First, in addition to having similar biological functions to those of parental MSCs, they are widely used because of their advantages of smaller size, lower immunogenicity, and easy accessibility (Lou et al., 2017). Second, the risk of tumor formation can be further reduced due to the absence of living cells present in the body. Finally, MSC-exos can also be used as carriers to synergize with drugs to treat hepatic fibrosis, in addition to directly intervening in hepatic fibrosis. However, there are also shortcomings in the use of MSC-exos for the treatment of liver fibrosis, such as their weak ability to target aHSCs, low exosome yield, low drug-carrying capacity, and low delivery efficiency, which need to be urgently addressed (Piffoux et al., 2018; Doyle and Wang, 2019; Cheng et al., 2021; Sun et al., 2021; Evers et al., 2022). In recent years, with the continuous and in-depth study of exosomes, researchers have found that MSC-exos can be used in combination with several anti-hepatic fibrosis drugs so that the anti-hepatic fibrosis ability can be further enhanced, which may be a major new strategy for the treatment of hepatic fibrosis by MSC-exos.

To systematically assess the efficacy of MSC-exos single administration and in combination with various anti-hepatic fibrosis drugs in treating hepatic fibrosis, a meta-analysis was conducted using an animal model. The analysis aimed to evaluate the impact of MSC-exos on pathological tissue changes in the liver, progression of hepatic fibrosis, and restoration of liver function. We further investigated the impact of MSC-exos’ source, extraction method, infusion method, type of the animal model, and modeling method on liver fibrosis treatment through subgroup analysis.

## 2 Methods

The detailed agreement is registered in PROSPERO. The registration number is CRD42024516199 (https://www.crd.york.ac.uk/PROSPERO/). This meta-analysis was carried out according to PRISMA guidelines (Supplementary Material S2).

### 2.1 Search strategies

**FIGURE 1:**
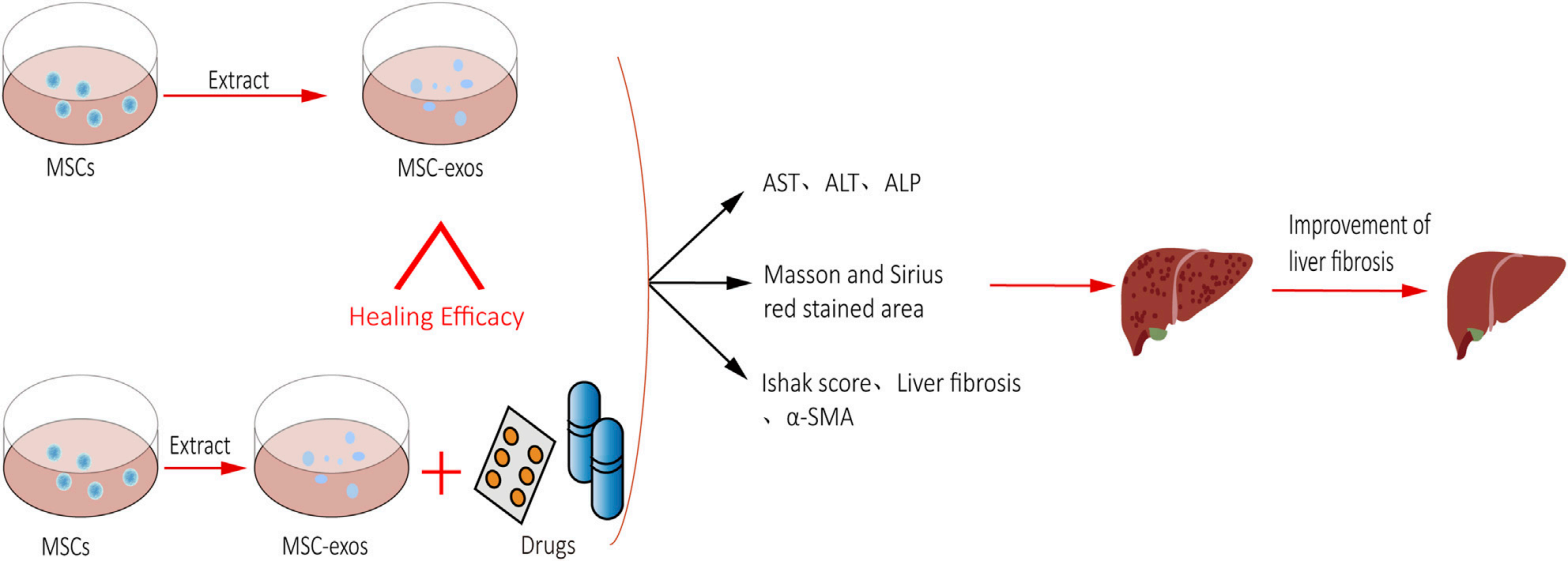
MSC-exos-drugs are superior to MSC-exos for treating liver fibrosis.

The sources retrieved were mainly from published literature on the Web of Science, PubMed, Embase, and Cochrane Library, which were published in English. We systematically searched for eligible studies from database creation to January 2024 using the keywords “mesenchymal stem cells,” “exosomes,” “extracellular vesicles,” and “liver fibrosis.” Details of the search are given in Supplementary Material S2. In addition, a further manual search of references was conducted to studies that met the inclusion criteria as a supplement (Figure 1).

### 2.2 Study selection

Two authors (Xiao-lei Zhou and Yan Xu) selected the literature that met the inclusion criteria by browsing the title, abstract, and keywords. We then obtained the full text after initial screening and subsequently evaluated the full text of potential studies to determine acceptability. Any differences were resolved by a consensus. The criteria for inclusion in the study were to meet the (population, intervention, comparison, outcomes, and study design) PICOS principle, as shown below.

### 2.3 Inclusion criteria

Subjects (P): animals with hepatic fibrosis.

Intervention (I): in MSC-exos single administration for liver fibrosis, the intervention was MSC-exos or placebo; in MSC-exos-drug administration for liver fibrosis, the intervention was MSC-exos-drugs or MSC-exos.

Comparison (C): in MSC-exos single administration for liver fibrosis, the control group was the placebo group; in MSC-exos-drug administration for liver fibrosis, the control group was the MSC-exos group.

Outcome (O): main results: ① hepatopathological histological changes: Sirius red-stained area and Masson-stained area. ② Evaluation of the degree of liver fibrosis: Ishak score, liver index, and α-smooth muscle actin (α-SMA). ③ Liver function: alanine aminotransferase (ALT), aspartate aminotransferase (AST), alkaline phosphatase (ALP), and hydroxyproline (Hyp).

Study design (S): only randomized controlled trials were included in this study.

### 2.4 Exclusion criteria

Conference abstracts, letters with duplicates, case reports, meta-analyses, reviews, literature not published in English, and studies with incomplete or unavailable data were excluded. In addition, studies that were not relevant to the article topic were excluded.

### 2.5 Data extraction

Two reviewers (Yan Xu and Xiao-lei Zhou) separately extracted the data for inclusion in the literature into Microsoft Excel spreadsheets and then summarized the data into tables. Any disagreements were resolved through careful discussion. When data were not available in the text, we used GetData Graph Digitizer version 2.25.0.32 software to extract the data from the graphs. The following information was extracted from the included literature: study characteristics (first author, year of publication, and country), animal characteristics (type of experimental animal and modeling method), intervention details (MSC-exos type, extraction method, exosome size, injection method, exosome dosage, and frequency of treatment), and primary outcome indicators.

### 2.6 Assessment of risk of bias in the included studies

Two reviewers (Xue-song Wang and Wen-ming Lu) independently assessed the risk of bias for each included study according to the Cochrane evaluation tool. Any disagreements were resolved by discussion between the two reviewers. The items assessed included the following: the generation of randomized outcomes, allocation concealment, blinding of subjects, investigators and outcome assessment, completeness of outcome information, selective reporting, and other sources of bias. Each item was classified as low risk, high risk, or unclear risk.

### 2.7 Statistical analyses

Review Manager 5.3 software was used to analyze the overall and subgroup treatment effects of MSC-exos single administration and MSC-exos-drug interventions on liver fibrosis. The data required for the meta-analysis were extracted directly from the primary literature or the graphs through the software. The pre-extracted means, standard deviations, and sample sizes were then entered into the analysis software. In our meta-analysis, standardized mean difference (SMD) was used to report continuous results. In addition, interstudy heterogeneity was analyzed using the I^2^ statistic, with I^2^ values <50% indicating low or moderate heterogeneity, and meta-analyses were performed using a fixed-effects model, with I^2^ values ≥50% indicating significant heterogeneity, using a random-effects model. The results with high heterogeneity between the two groups were evaluated using sensitivity analyses or subgroup analyses (sensitivity analyses were performed using Stata/MP 17).

## 3 Results

### 3.1 Results of the search

**FIGURE 2:**
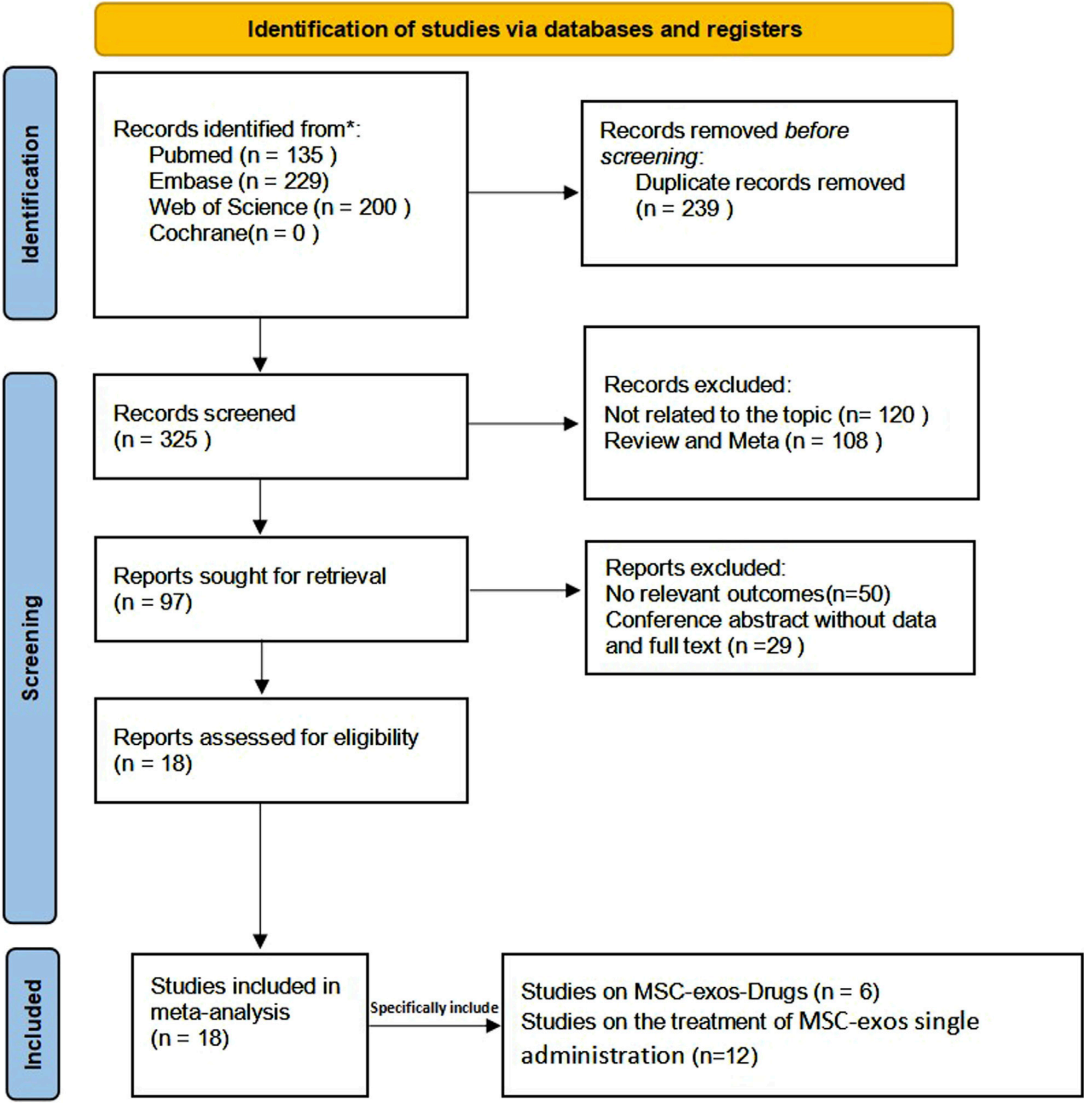
Literature selection and inclusion process.

The system searched four databases and retrieved a total of 564 papers, of which 135 were retrieved from PubMed, 229 from Embase, 200 from the Web of Science, and 0 from the Cochrane Library. After eliminating duplicates, 325 articles remained. After reading the title abstracts, 228 articles were excluded, of which 108 articles were from previous reviews and meta-analyses, and 120 articles were from studies unrelated to the topic; 97 potentially eligible articles were included. However, when the full texts were reviewed, 29 articles were conference abstracts, and 50 articles had no relevant outcome indicators. Therefore, 18 articles were included in our meta-analysis, of which 12 involved the MSC-exos single administration and six involved the use of MSC-exos-drugs. The detailed screening and inclusion process is shown in Figure 2.

### 3.2 Characteristics of the studies

Of the 18 articles included, one each was from Japan and India, eight were from China, four were from Egypt, and the remaining four were from South Korea and Iran. The sample sizes of all the studies ranged from 6 to 40, and they were published between 2013 and 2023. The main extraction methods used for MSC-exos were ultracentrifugation *versus* kit extraction, and the main infusion methods used were tail vein injection and intraperitoneal injection. Tables 1, 2 list the detailed characteristics of the included studies (differentiated according to MSC-exos single administration *versus* MSC-exos-drugs).

**TABLE 1:** Summary of animal studies of MSC-exos single administration for the treatment of liver fibrosis.

Included studies	Year	Country	Species	Modeling methods	Origin of exosomes	Isolation technique	Average diameter	Exosomes infusion (method and dose)	Experimental grouping (N)∗	Therapy cycle	Available outcomes
Tan et al. (2022)	2022	China	Female BALB/c mice	CCl_4_ Injection	hUC-MSCs	Ultracentrifugation method	100 nm	Tail vein injection	Control group:6 Treatment group:6	Twice a week for 2 weeks	Sirius red
Zhang et al. (2022)	2022	China	Male SD rats	CCl_4_ Injection	BM-MSCs	Ultracentrifugation method	——	Tail vein injection (100 μg, 200 μg, and 400 μg)	Control group:5 Treatment group:5	Twice a week for 4 weeks	AST, ALT, ALB, and Masson-stained
Zhang et al. (2023a)	2023	China	Male C57BL/6 J mice	CCl_4_ Injection	ADSCs	Ultracentrifugation method	50–100 nm	Tail vein injection (250 μg)	Control group:6 Treatment group:6	Twice a week for 4 weeks	Sirius red, Masson-stained, ALT, AST, and Ishak
Kim et al. (2020)	2020	Korea	Male C57BL/6 mice	CCl_4_ Injection	TMSCs	Ultracentrifugation method	50–100 nm	Tail vein injection (150 mg)	Control group:5 Treatment group:6	Three times for 2 weeks	AST, ALT, α-SMA, and Sirius red
Rong et al. (2019)	2019	China	Female SD rats	CCl_4_ Injection	BM-MSCs	Ultracentrifugation method	——	Tail vein injection (250 mg)	Control group:12 Treatment group:12	One time for 4 weeks	Masson-stained, Ishak, Hyp, ALT, AST, ALP, and α-SMA
Niknam et al. (2023)	2023	Iran	Male BALB/C mice	CCl_4_ Injection	hUC-MSCs	The exosome isolation kit (PEG precipitation)	——	Intraperitoneal injection (80 µg)	Control group:5 Treatment group:5	Once a week for 3 weeks	Masson-stained, Sirius red, α-SMA, ALT, AST, ALP, and ALB
Gan et al. (2023)	2023	China	Male C57BL/6 mice	CCl_4_ Injection	ADSCs	ExoQuick reagent (PEG precipitation)	——	Tail vein injection (40 µg)	Control group:6 Treatment group:6	Twice a week for 6 weeks	α-SMA, ALT, AST, and Masson-stained
Gupta et al. (2022)	2022	India	Female mice	CCl_4_ Injection	ADMSCs hUC-MSCs	Ultracentrifugation method	——	Tail vein injection (250 μg)	Control group:5 Treatment group:5	Once in all	Masson-stained, Sirius red, and α-SMA
Li et al. (2013)	2013	China	Kunmingbai strains mice	CCl_4_ Injection	hUC-MSCs	Ultracentrifugation method	40–100 nm	Liver lobe injection (250 mg)	Control group:6 Treatment group:6	Once in all	ALT, AST, and Masson-stained
Wu et al. (2022)	2022	China	Male C57/BL6 mice	DEN/CCl_4_ Injection	ADSCs	Ultracentrifugation method	30–150 nm	Tail vein injection	Control group:5 Treatment group:5	Three times for 2 weeks	Liver index, Ishak, Sirius red, ALT, AST, and α-SMA
Han et al. (2020)	2020	Korea	Male C57BL/6 mice	TAA Injection	ADSCs	Tangential flow filtration	94.2 ± 4.7 nm	Tail vein injection	Control group:5 Treatment group:5	Once in all	Masson-stained, α-SMA, and Hyp
Ohara et al. (2018)	2018	Japan	Male SD rats	CCl_4_ Injection	AMSCs	Ultracentrifugation method	80–110 nm	Penile vein injection	Control group:11 Treatment group:11	Once in all	Masson-stained and α-SMA

^a^ Control group refers to the group treated with PBS for liver fibrosis. The treatment group refers to the use of MSC-exos for the treatment of liver fibrosis.

**TABLE 2:** Summary of animal studies of MSC-exos-drugs for the treatment of liver fibrosis.

Included studies	Year	Country	Species	Modeling methods	Origin of exosomes	Isolation technique	Average diameter	Type of drug	Experimental grouping (N)∗	Exosomes (method and dose)	Drug taking (method and dose)	Therapy cycle	Available Outcomes
Azizsoltani et al. (2023)	2023	Iran	Male BALB/C mice	CCl_4_ injection	hUC-MSCs	An AnaCell kit (PEG precipitation)	71.65 ± 6.78 nm	Obeticholic acid	Control group:5 Treatment group:5	Intraperitoneal injection (100 µg)	Intraperitoneal injection (5 mg/kg)	Five times for 3 weeks	Masson-stained, Sirius red, ALT, and AST
Didamoony et al. (2023)	2023	Egypt	Male Wistar rats	DEN injection	BM-MSCs	Ultracentrifugation method	——	Rupatadine	Control group:6 Treatment group:6	Tail vein injection	Oral (4 mg/kg)	Once a week for 4 weeks	ALT, AST, liver index, and Masson-stained
Shiha et al. (2020)	2020	Egypt	Male Wistar rats	CCl_4_ injection	MSCs	Ultracentrifugation method	——	Nilotinib	Control group:10 Treatment group:10	Tail vein injection	Gavage (20 mg/kg)	Once a day for 5 weeks	ALT and Masson-stained
Fang and Liang (2021)	2021	China	Male mice	CCl_4_ injection	ADSCs	ExoEasy Maxi Kit (spin-column filtration)	——	Quercetin	Control group:3 Treatment group:3	Tail vein injection	Intraperitoneal injection	——	ALT, AST, and liver index
Ashour et al. (2022)	2022	Egypt	Male SD rats	CCl_4_ injection	BM-MSCs	Ultracentrifugation method	89.4 ± 7.5 nm	Luteolin	Control group:7 Treatment group:7	Intraperitoneal injection (81–99 µg)	Intraperitoneal injection (1.4 mg/kg)	——	ALT, AST, liver index, and Masson-stained
Ellakany et al. (2023)	2023	Egypt	Male mice	Schistosomiasis	BM-MSCs	Ultracentrifugation method	40–80 nm	Praziquantel	Control group:20 Treatment group:20	Intraperitoneal injection	——	——	Masson-stained

^a^ Control group refers to the group treated with MSC-exos for liver fibrosis. Treatment group refers to the use of MSC-exos–drugs for the treatment of liver fibrosis.

### 3.3 Risk of bias assessment

**FIGURE 3:**
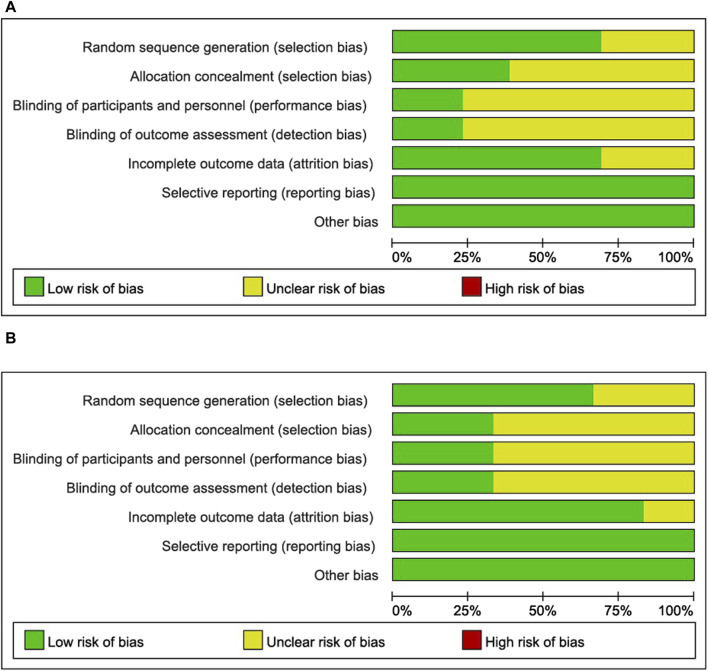
Risk of bias graph: review authors’ judgments about each risk of bias item presented as percentages across all included studies. **(A)**: MSC-exos single administration for the treatment of liver fibrosis. **(B)**: Use of MSC-exos-drugs for the treatment of hepatic fibrosis.

**FIGURE 4:**
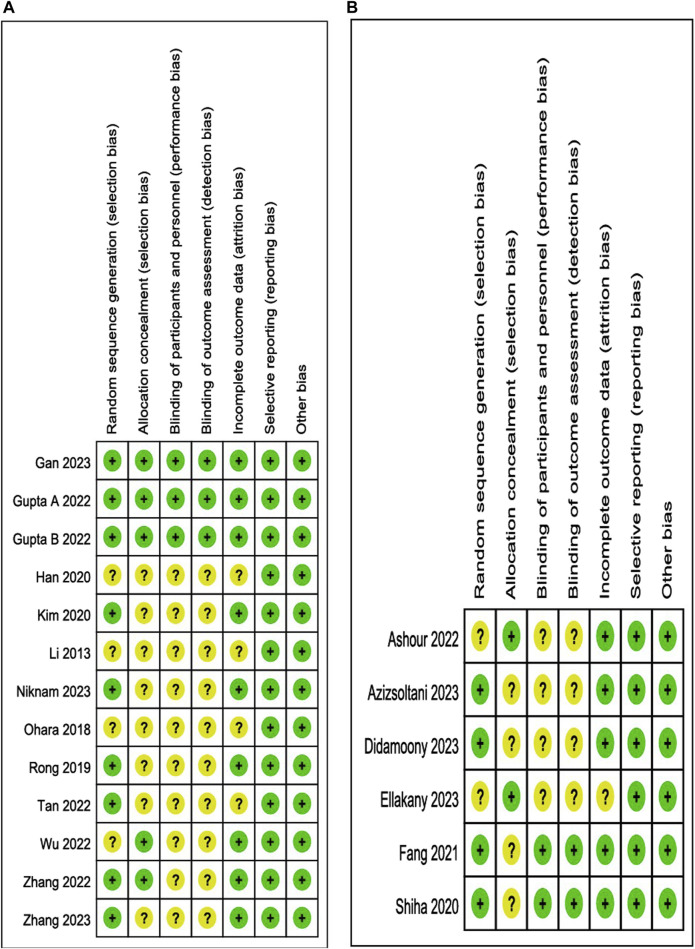
Risk of bias summary: review authors’ judgments about each risk of bias item for each included study. **(A)**: MSC-exos single administration for the treatment of liver fibrosis. **(B)**: Single administration of MSC-exos for the treatment of liver fibrosis.

The results of the risk of bias and methodological applicability assessment of the included studies are shown in Figures 3, 4. A total of 12 articles (13 studies) on the single administration of MSC-exos and six articles (six studies) on MSC-exos-drugs were included in this paper. In the literature related to the single administration of MSC-exos for liver fibrosis, nine studies had a low risk of bias (Rong et al., 2019; Kim et al., 2020; Gupta et al., 2022; Tan et al., 2022; Zhang et al., 2022; Zhang ZL. et al., 2023; Gan et al., 2023; Niknam et al., 2023), and four studies did not mention randomized outcome generation (unclear risk of bias) (Li et al., 2013; Ohara et al., 2018; Han et al., 2020; Wu et al., 2022). Five studies mentioned allocation concealment (Gupta et al., 2022; Wu et al., 2022; Zhang et al., 2022; Gan et al., 2023), and eight studies did not mention allocation concealment (risk of bias not yet known) (Li et al., 2013; Ohara et al., 2018; Rong et al., 2019; Han et al., 2020; Kim et al., 2020; Tan et al., 2022; Zhang ZL. et al., 2023; Niknam et al., 2023). For blinding of outcome assessment, three studies were with low risk (Gupta et al., 2022; Gan et al., 2023), and 10 studies were not reported (risk of bias not known) (Li et al., 2013; Ohara et al., 2018; Rong et al., 2019; Han et al., 2020; Kim et al., 2020; Tan et al., 2022; Wu et al., 2022; Zhang et al., 2022; Zhang ZL. et al., 2023; Niknam et al., 2023). Most of the studies had complete information on the results (low risk of bias), and only four studies reported no mention (risk of bias not yet known) (Li et al., 2013; Ohara et al., 2018; Han et al., 2020; Tan et al., 2022). All studies were free of selective reporting bias and other biases. Funnel plot analyses were not performed due to an insufficient number of included studies.

However, in the literature related to MSC-exos-drugs, four studies had a low risk of bias (Shiha et al., 2020; Fang and Liang, 2021; Azizsoltani et al., 2023; Didamoony et al., 2023), and two studies did not mention randomized outcome generation (the risk of bias was unclear) (Ashour et al., 2022; Ellakany et al., 2023). Two studies mentioned allocation concealment (Ashour et al., 2022; Ellakany et al., 2023), and four studies did not mention allocation concealment (risk of bias unclear) (Shiha et al., 2020; Fang and Liang, 2021; Azizsoltani et al., 2023; Didamoony et al., 2023). For blinding of outcome assessment, the two studies were low risk (Shiha et al., 2020; Fang and Liang, 2021), and four studies were not reported (unclear risk of bias) (Ashour et al., 2022; Azizsoltani et al., 2023; Didamoony et al., 2023; Ellakany et al., 2023). Most studies had complete information on the results (low risk of bias), and only one study reported no mention (risk of bias not yet known) (Ellakany et al., 2023). All studies were free from selective reporting bias and other biases. Funnel plot analysis was also not performed due to an insufficient number of included studies.

### 3.4 Meta-analysis

Eighteen eligible articles were included in the meta-analysis, of which 12 involved the MSC-exos single administration and 6 involved the use of MSC-exos-drugs. On the one hand, the efficacy of MSC-exos alone in the treatment of liver fibrosis was assessed in terms of the pathological histological changes in the liver (Masson-stained area and Sirius red-stained area), the progression of hepatic fibrosis (Ishak score, liver index, and α-SMA), and the liver function (ALT, AST, ALP, and Hyp). On the other hand, MSC-exos-drugs were analyzed as superior to the single administration of MSC-exos from different perspectives.

## 4 Meta-analysis of MSC-exos single administration in the treatment of liver fibrosis

### 4.1 The single administration of MSC-exos significantly inhibits collagen deposition and fibroproliferation

#### 4.1.1 Masson-stained area

**FIGURE 5:**
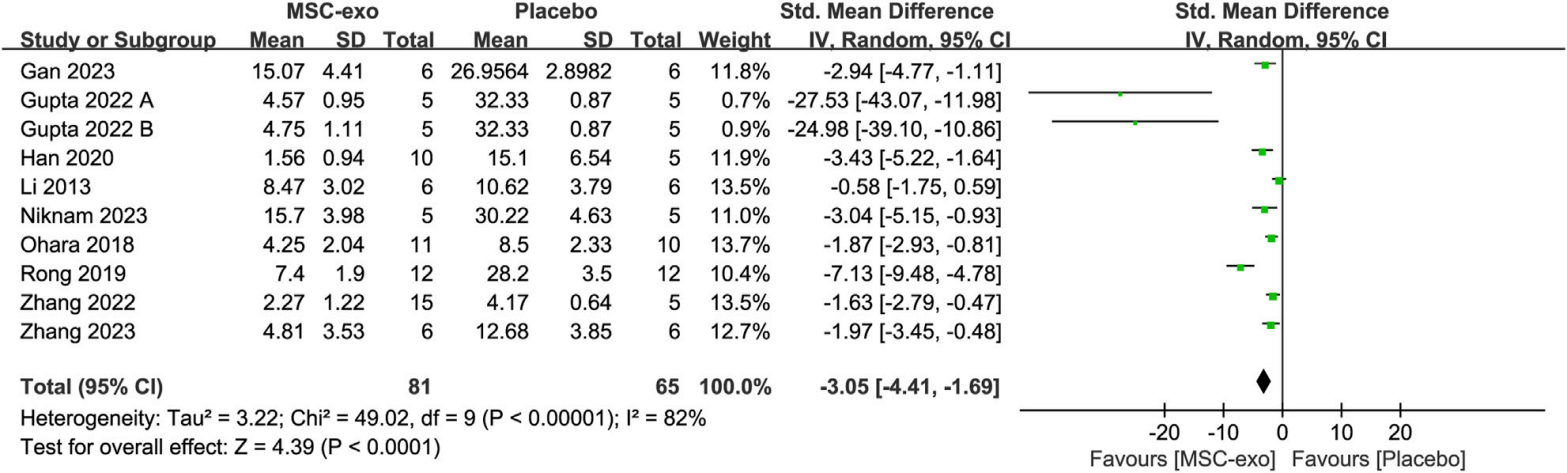
Forest plot of the Masson-stained area.

The Masson-stained area was reported in eight studies for 69 animals in the MSC-exos group and 53 animals in the placebo group. Their combined results showed that the Masson-stained area was significantly lower in the MSC-exos group than in the placebo group [SMD = −3.05; 95% CI = (−4.41, −1.69); *p* < 0.0001; heterogeneity test I^2^ = 82%; *p* < 0.00001]. Subgroup analyses were performed to explore the effects of various factors on the Masson-stained area in the treatment of liver fibrosis with MSC-exos. (Figure 5).

#### 4.1.2 Subgroups of the MSC-exos source for Masson-stained area level

To investigate the effect of the infusion of different sources of MSC-exos on the Masson-stained area, we performed a subgroup analysis of MSC-exos sources. The combined results showed that the MSC-exos group significantly reduced Masson-stained area compared to the placebo group [SMD = −3.05; 95% CI = (−4.41, −1.69); *p* < 0.0001; heterogeneity test I^2^ = 82%; *p* < 0.00001]. Random-effects model subgroup analyses showed that ADSC-exos [SMD = −3.24; 95% CI = (−5.44, −1.03); *p* = 0.03; heterogeneity test I^2^ = 87%; *p* = 0.004] and AMSC-exos [SMD = −1.87; 95% CI = (−2.93, −0.81); *p* = 0.0006] significantly reduced the Masson-stained area compared with the placebo group. However, there was no statistical difference in either the hUC-MSC-exos group [SMD = −3.55; 95% CI = (−7.82, 0.71); *p* = 0.10; heterogeneity test I^2^ = 87%; *p* = 0.0006] or the BM-MSC-exos group [SMD = −4.28; 95% CI = (−9.67, 1.11); *p* = 0.12; heterogeneity test I^2^ = 94%; *p* < 0.0001].

#### 4.1.3 Subgroups of experimental animals for Masson-stained area level

**FIGURE 6:**
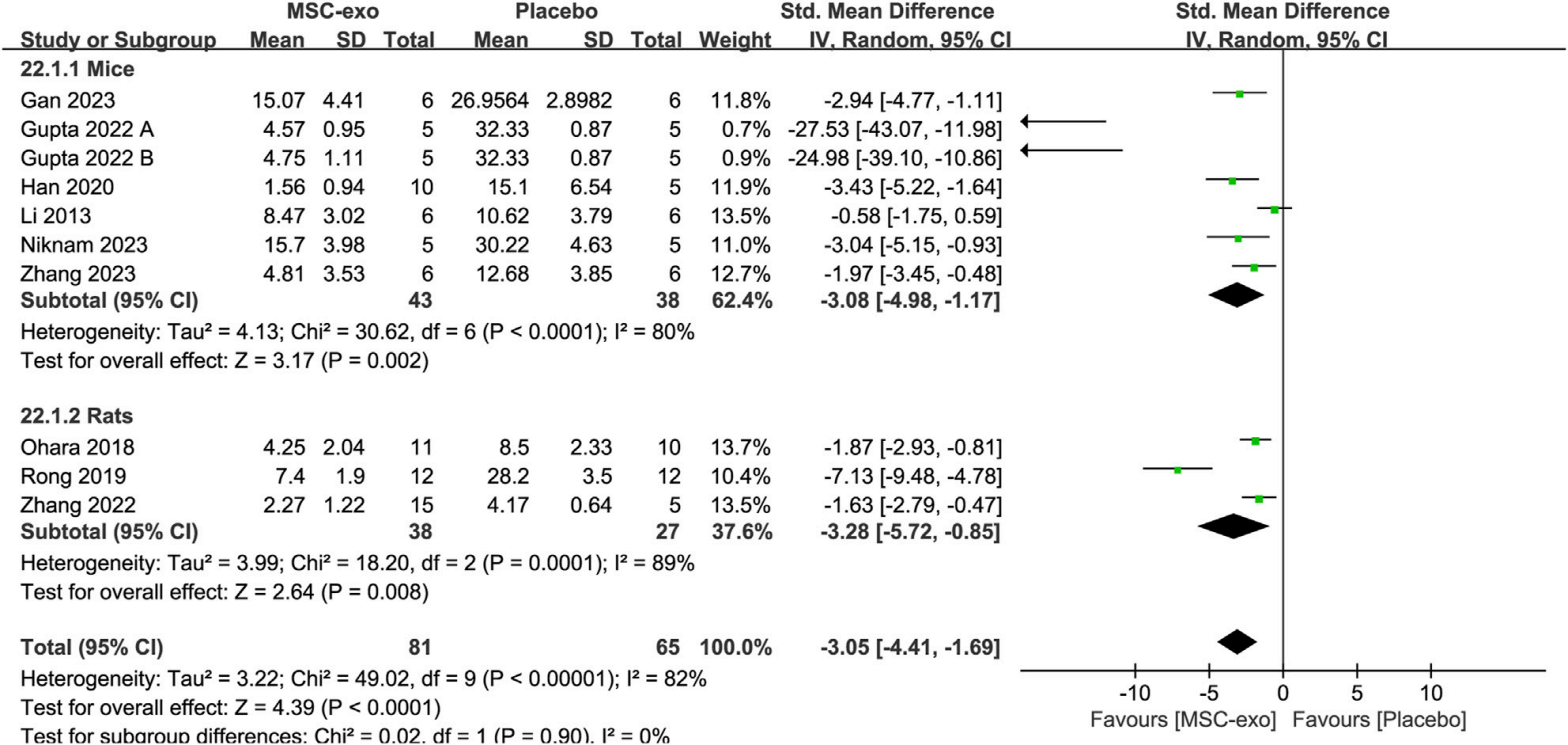
Subgroups of experimental animals for the Masson-stained area.

In the included literature, the experimental animals mainly consisted of mice and rats. Random-effects model subgroup analyses showed that MSC-exos significantly reduced the Masson-stained area in mice [SMD = −3.08; 95% CI = (−4.98, −1.17); *p* = 0.002; heterogeneity test I^2^ = 80%; *p* < 0.0001] and rats [SMD = −3.28; 95% CI = (−5.72, −0.85); *p* = 0.008; heterogeneity test I^2^ = 89%; *p* = 0.0001] (Figure 6).

### 4.1.4 Sirius red-stained area

**FIGURE 7:**
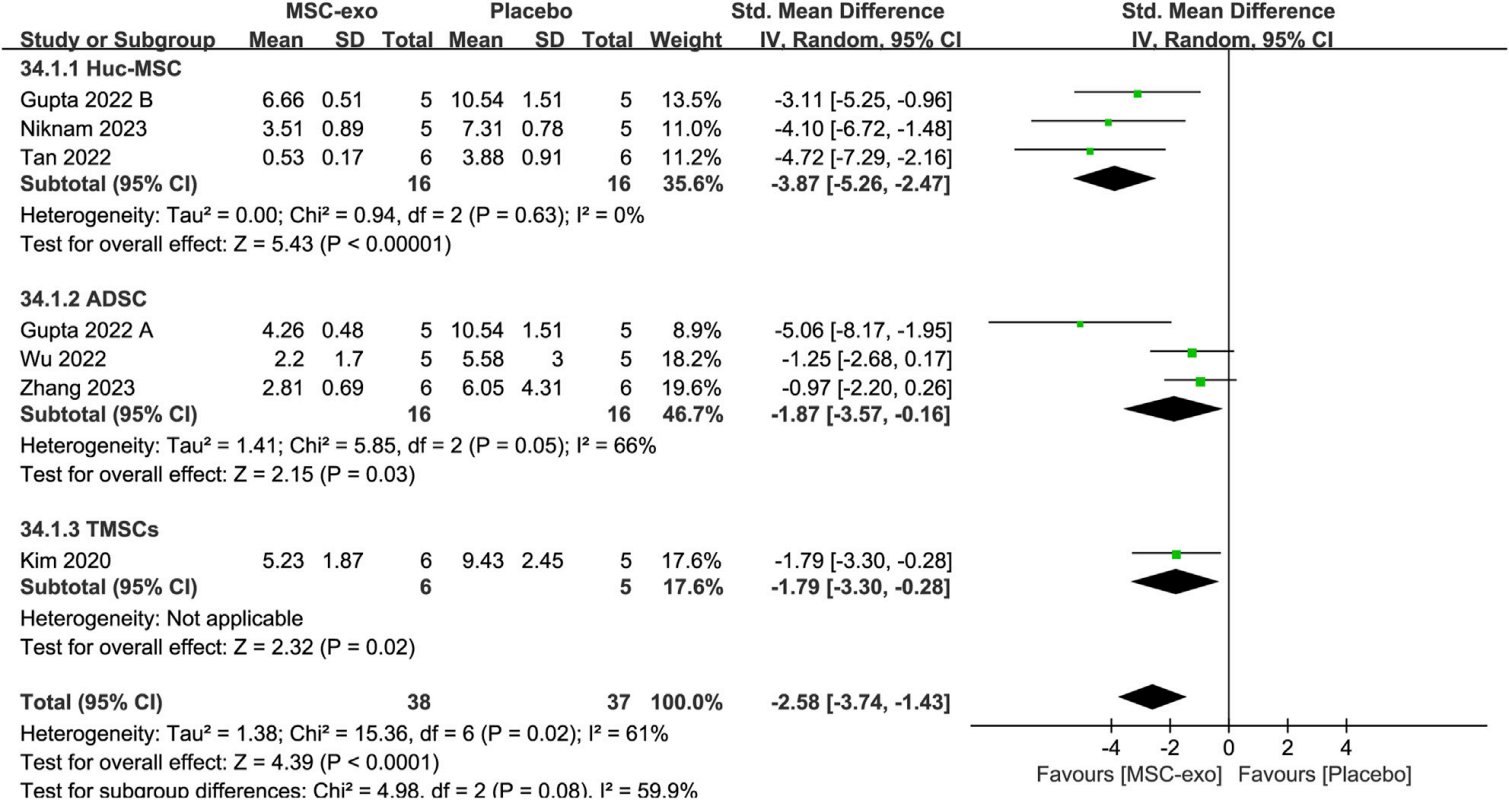
Subgroups of the MSC-exos source for the Sirius red-stained area.

Six studies reported the Sirius red-stained area in 32 animals in the MSC-exos group and 32 animals in the control group. The combined results showed that compared to the placebo group, the Sirius red-stained area in the MSC-exos group was significantly decreased [SMD = −2.58; 95% CI = (−3.74, −1.43); *p* < 0.0001; heterogeneity test I^2^ = 61%; *p* = 0.02]. Subgroup analyses were performed to make the conclusions more accurate. For the MSC-exos source subgroup of Sirius red-stained area levels, random-effects model subgroup analyses showed that the use of hUC-MSC-exos [SMD = −3.87; 95% CI = (−5.26, −2.47); *p* < 0.00001; heterogeneity test I^2^ = 0%; *p* = 0.63], ADSC-exos [SMD = −1.87; 95% CI = (−3.57, −0.16); *p* = 0.03; heterogeneity test I^2^ = 66%; *p* = 0.05], or TMSC-exos treatment [SMD = −1.79; 95% CI = (−3.30, −0.28); *p* = 0.02] significantly reduced the Sirius red-stained area (Figure 7).

### 4.2 The single administration of MSC-exos significantly delays the progression of liver fibrosis

#### 4.2.1 α-SMA

**FIGURE 8:**
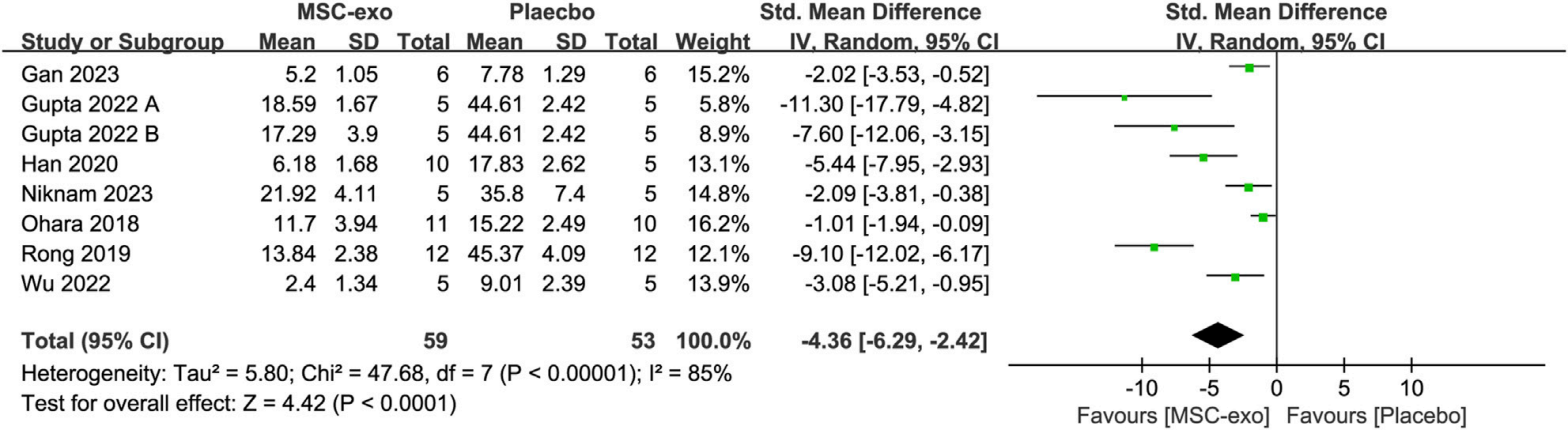
Forest plot of α-SMA.

α-SMA was measured in eight studies with 59 animals in the MSC-exos group and 53 animals in the placebo group. Their combined results showed that the α-SMA level was significantly lower in the MSC-exos group than in the placebo group [SMD = −4.36; 95% CI = (−6.29, −2.42); *p* < 0.0001; heterogeneity test I^2^ = 85%; *p* < 0.00001]. In the treatment of liver fibrosis with MSC-exos, a subgroup analysis was performed to explore the effects of various factors on α-SMA (Figure 8).

#### 4.2.2 Subgroups of MSC-exos source for α-SMA level

To investigate the effect of the infusion of different sources of MSC-exos on ALT, we performed a subgroup analysis of MSC-exos sources. Random-effects model subgroup analysis showed that treatment of hepatic fibrosis with ADSC-exos resulted in a decrease in the α-SMA level [SMD = −4.23; 95% CI = (−6.69, −1.77); *p* = 0.0008; heterogeneity test I^2^ = 74%; *p* = 0.01], whereas the results in the hUC-MSC-exos group were not statistically significant [SMD = −4.45; 95% CI = (−9.79, 0.89); *p* = 0.1; heterogeneity test I^2^ = 80%; *p* = 0.02] (Supplementary Figure S1 A).

#### 4.2.3 Subgroups of experimental animals for α-SMA level

In the included literature, the experimental animals mainly included mice and rats. Random-effects model subgroup analyses revealed that the MSC-exos significantly reduced the α-SMA index with mice as the experimental animal [SMD = −4.04; 95% CI = (−5.90, −2.19); *p* < 0.001; heterogeneity test I^2^ = 70%; *p* = 0.005]. In contrast, the results for the subgroup with rats as the experimental animals were not statistically significant [SMD = −4.93; 95% CI = (−12.85, 2.99); *p* = 0.22; heterogeneity test I^2^ = 96%; *p* < 0.00001] (Supplementary Figure S1 B).

#### 4.2.4 Subgroups of MSC-exos extraction methods for α-SMA level

The extraction methods used in the eight studies we included mainly included ultracentrifugation, kit extraction, and TFF. Random-effects model subgroup analyses revealed that for the extraction of α-SMA levels using ultracentrifugation [SMD = −5.82; 95% CI = (−9.52, −2.13); *p* = 0.002; heterogeneity test I^2^ = 90%; *p* < 0.00001] or kit extraction [SMD = −2.06; 95% CI = (−3.18, −0.93); *p* = 0.0004; heterogeneity test I^2^ = 0%; *p* = 0.95], the α-SMA lowering effect was better in the MSC-exos group than in the placebo group (Supplementary Figure S1 C).

### 4.3 The single administration of MSC-exos significantly improves liver function

#### 4.3.1 AST

Serum AST was reported in eight studies for 57 animals in the MSC-exos group and 47 animals in the placebo group. The combined results showed a significant reduction in the AST index in the MSC-exos group compared to that in the placebo group [SMD = −2.99; 95% CI = (−4.29, −1.70); *p* < 0.00001; heterogeneity test I^2^ = 74%; *p* = 0.0003]. A subgroup analysis was performed to investigate the effect of various factors on serum AST in the treatment of hepatic fibrosis with MSC-exos.

#### 4.3.2 Subgroups of the MSC-exos source at the AST level

To explore the effect of the infusion of different sources of MSC-exos on AST, we analyzed the MSC-exos source subgroup. The combined results showed that the AST index was significantly lower in the MSC-exos group than in the placebo group [SMD = −2.99; 95% CI = (−4.29, −1.70); *p* < 0.00001; heterogeneity test I^2^ = 74%; *p* = 0.0003]. Random-effects model subgroup analyses revealed that the AST index was significantly lower in both the hUC-MSC-exos [SMD = −3.46; 95% CI = (−4.98, −1.93); *p* < 0.00001; heterogeneity test I^2^ = 0%; *p* = 0.73] and ADSC-exos groups [SMD = −2.77; 95% CI = (−5.27, −0.27); *p* = 0.03; heterogeneity test I^2^ = 77%; *p* = 0.01] compared with that in the placebo group. However, there was no statistical difference in the BM-MSC-exos group [SMD = −4.13; 95% CI = (−8.52, 0.25); *p* = 0.06; heterogeneity test I^2^ = 92%; *p* = 0.0004] (Supplementary Figure S2).

#### 4.3.3 Subgroups of the MSC-exos extraction mode at the AST level

**FIGURE 9:**
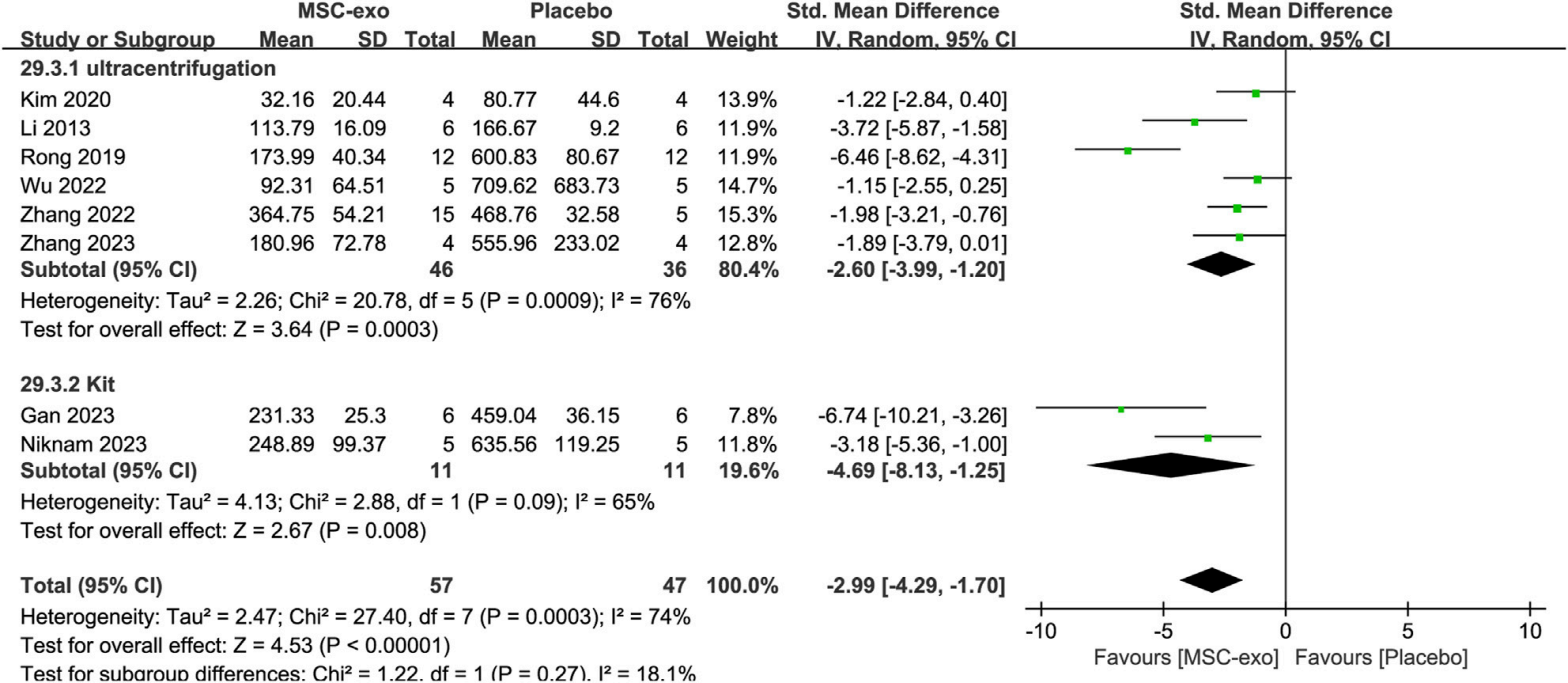
Subgroups of MSC-exos extraction methods for AST level.

In the eight studies we included, the extraction methods mainly included ultracentrifugation *versus* kit extraction. Random-effects model subgroup analysis revealed that when ultracentrifugation [SMD = −2.99; 95% CI = (−4.41, −1.57); *p* < 0.0001; heterogeneity test I^2^ = 72%; *p* = 0.003] or kit extraction [SMD = −4.69; 95% CI = (−8.13, −1.25); *p* = 0.008; heterogeneity test I^2^ = 65%; *p* = 0.09] was used, the ASTs were significantly reduced, and all of them were superior to those of the placebo group (Figure 9).

#### 4.3.4 Subgroups of experimental animals for AST levels

In the included literature, experimental animals mainly consisted of mice *versus* rats. Random-effects model subgroup analysis showed that the AST index was significantly reduced in the MSC-exos group when using mice as the experimental animals [SMD = −2.55; 95% CI = (−3.85, −1.25); *p* = 0.0001; heterogeneity test I^2^ = 61%; *p* = 0.02]). In contrast, the results for the subgroup with rats as the experimental animals were not statistically significant [SMD = −4.13; 95% CI = (−8.52, 0.25); *p* = 0.06; heterogeneity test I^2^ = 92%; *p* = 0.0004].

#### 4.3.5 ALT

**FIGURE 10:**
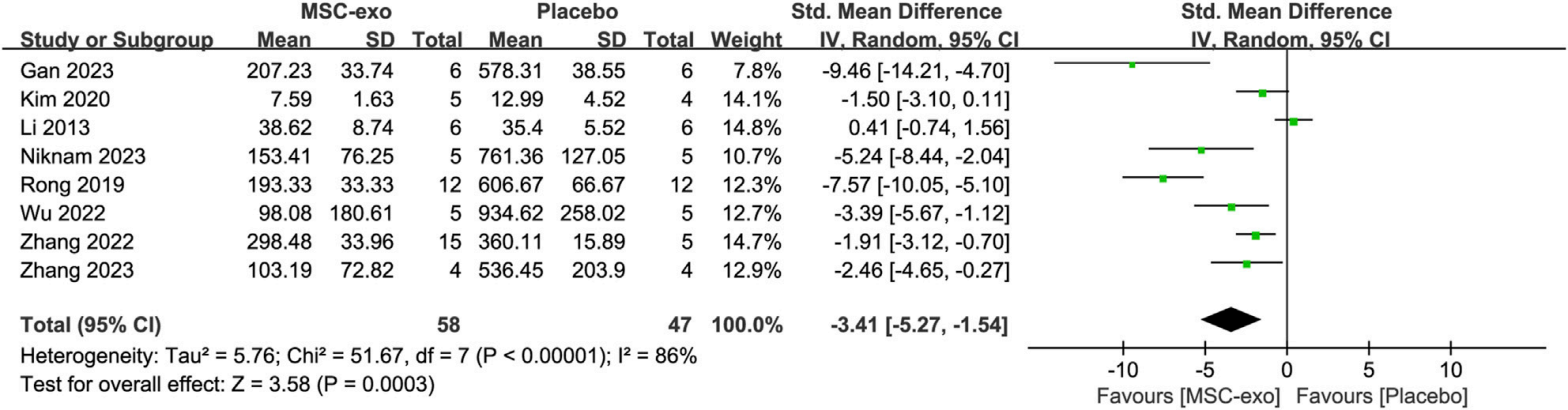
Forest plot of ALT.

Serum ALT was reported in eight studies for 58 animals in the MSC-exos group and 47 animals in the placebo group. Their combined results showed that the ALT level was significantly lower in the MSC-exos group than in the placebo group [SMD = −3.41; 95% CI = (−5.27, −1.54); *p* = 0.0003; heterogeneity test I^2^ = 86%; *p* < 0.00001]. Subgroup analysis was performed to explore the effect of various factors on ALT in the treatment of liver fibrosis with MSC-exos (Figure 10).

#### 4.3.6 Subgroups of the MSC-exos source at the ALT level

To investigate the effect of the infusion of different sources of MSC-exos on ALT, we performed a subgroup analysis of MSC-exos sources. Random-effects model subgroup analysis showed that the effects of treatment with other sources of MSC-exos were not statistically significant, except for the effects of the treatment of hepatic fibrosis with ADSC-exos, which resulted in a decrease in ALT [SMD = −4.38; 95% CI = (−7.42, −1.34); *p* = 0.005; heterogeneity test I^2^ = 71%; *p* = 0.03].

#### 4.3.7 Subgroups of the MSC-exos extraction method at the ALT level

In the eight studies we included, the extraction methods mainly included ultracentrifugation *versus* kit extraction. Random-effects model subgroup analysis showed that after extraction via ultracentrifugation [SMD = −2.56; 95% CI = (−4.42, −0.69); *p* = 0.007; heterogeneity test I^2^ = 87%; *p* < 0.00001] or extraction via a kit [SMD = −6.97; 95% CI = (−11.03, −2.90); *p* = 0.0008; heterogeneity test I^2^ = 52%; *p* = 0.15], the reduction in ALT in the MSC-exos group was superior to that in the placebo group (Supplementary Figure S3 A).

#### 4.3.8 Subgroups of experimental animals for ALT levels

In the included literature, the experimental animals mainly included mice vs rats. Random-effects model subgroup analyses showed that the MSC-exos group significantly reduced ALT metrics with mice as the experimental animal [SMD = −2.98; 95% CI = (−5.11, −0.86); *p* = 0.006; heterogeneity test I^2^ = 83%; *p* < 0.0001]. In contrast, the results for the subgroup with rats as the experimental animals were not statistically significant [SMD = −4.63; 95% CI = (−10.18, 0.91); *p* = 0.10; heterogeneity test I^2^ = 94%; *p* < 0.0001] (Supplementary Figure S3 B).

In addition, the combined results of the secondary outcome indicators also demonstrated that MSC-exos were more efficacious than placebo treatment for hepatic fibrosis, but subgroup analyses were not performed due to the insufficient number of included studies (Table 3).

**TABLE 3:** Secondary outcome indicators comparing the efficacy of MSC-exos to that of the placebo.

Outcomes	Number of animals	Std. mean difference (95% CI)	Test for effect (*p*-value)	Heterogeneity, I^2^ (%)
Liver index	30	2.11 (−2.06, 6.27)	*p* = 0.32	I^2^ = 91
Ishak	46	−3.52 (−6.00, −1.04)	*p* = 0.005	I^2^ = 82
ALP	42	−3.35 (−5.37, −1.33)	*p* = 0.001	I^2^ = 70
Hyp	67	−3.09 (−5.24, −0.93)	*p* = 0.005	I^2^ = 80

### 4.4 Meta-analysis of MSC-exos-drugs for liver fibrosis

MSC-exos have both overcome the shortcomings of MSCs and demonstrated their unique advantages in the treatment of liver fibrosis. In recent years, with the continuous exploration of researchers, MSC-exos-drugs have been found to be even more effective than MSC-exos single administration. Therefore, to obtain a more comprehensive understanding of the efficacy of MSC-exos-drugs for liver fibrosis treatment, this paper included six studies of literature related to MSC-exos-drugs to explore where MSC-exos-drugs are more advantageous.

The efficacy of MSC-exos single administration and MSC-exos-drugs was analyzed in a model of hepatic fibrosis through six included studies. Combined analyses revealed that MSC-exos-drugs were superior to MSC-exos alone in terms of Masson-stained area, liver index, ALT, and AST, while subgroup analyses revealed less stable results (Table 4).

**TABLE 4:** Meta-analysis comparing the efficacy of MSC-exos-drugs with that of MSC-exos single administration.

Outcomes	Way of analysis	Subgroup	Specific classification	Std. mean difference (95% CI)	Test for effect (*p*-value)	Heterogeneity, I^2^ (%)
Masson-stained area	Combined results	——	——	−5.88 (−9.52, −2.25)	*p* = 0.002	I^2^ = 89
	Subgroup analysis	Infusion modalities	Tail vein injection	−2.86 (−6.85, 1.14)	*p* = 0.16	I^2^ = 75
			Intraperitoneal injection	−7.97 (−12.90, −3.04)	*p* = 0.002	I^2^ = 82
		Experimental animals	Mice	−10.04 (−17.45, −2.63)	*p* = 0.008	I^2^ = 82
			Rats	−3.42 (−6.35, −0.49)	*p* = 0.02	I^2^ = 81
Liver index	Combined results	——	——	−2.13 (−3.12, −1.15)	*p* = 0.51	I^2^ = 0
	Subgroup analysis	Lack of sufficient volume of data for subgroup analysis				
AST	Combined results	——	——	−2.25 (−3.98, −0.53)	*p* = 0.01	I^2^ = 69
	Subgroup analysis	Extraction method	Ultracentrifugation	−3.91 (−9.96, 2.13)	*p* = 0.2	I^2^ = 90
			Kits	−1.69 (−2.98, −0.40)	*p* = 0.01	I^2^ = 0
		Experimental animals	Mice	−1.69 (−2.98, −0.40)	*p* = 0.01	I^2^ = 0
			Rats	−3.91 (−9.96, 2.13)	*p* = 0.2	I^2^ = 90
		Infusion modalities	Tail vein injection	−4.26 (−9.76, 1.24)	*p* = 0.13	I^2^ = 0
			Intraperitoneal injection	−1.30 (−2.23, −0.37)	*p* = 0.006	I^2^ = 69
ALT	Combined results	——	——	−2.45 (−3.91, −0.99)	*p* = 0.001	I^2^ = 71
	Subgroup analysis	Infusion modalities	Tail vein injection	−2.11 (−4.14, −0.07)	*p* = 0.04	I^2^ = 74
			Intraperitoneal injection	−3.06 (−5.36, −0.75)	*p* = 0.009	I^2^ = 65
		Extraction method	Ultracentrifugation	−3.38 (−6.19, −0.57)	*p* = 0.02	I^2^ = 84
			Kits	−1.54 (−2.78, −0.29)	*p* = 0.02	I^2^ = 0
		Experimental animals	Mice	−1.54 (−2.78, −0.29)	*p* = 0.02	I^2^ = 0
			Rats	−3.38 (−6.19, −0.57)	*p* = 0.02	I^2^ = 84

### 4.5 Sensitivity analysis

**FIGURE 11:**
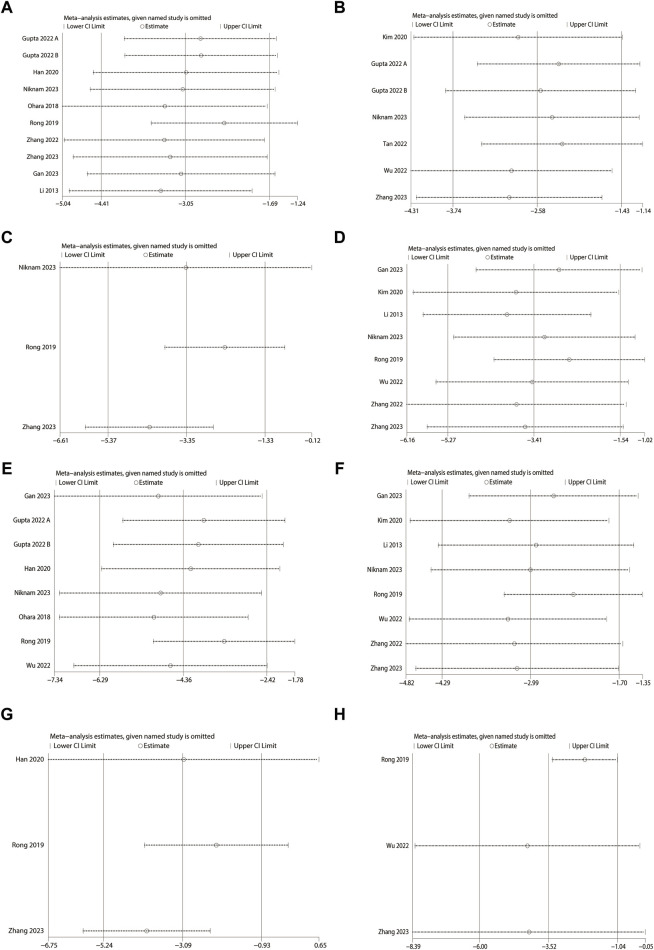
Sensitivity analysis (MSC-exos single administration). **(A)** Masson-stained area. **(B)** Sirius red-stained area. **(C)** Alkaline phosphatase (ALP). **(D)** Alanine aminotransferase (ALT). **(E)** α-smooth muscle actin (α-SMA). **(F)** Aspartate aminotransferase (AST). **(G)** Hydroxyproline (Hyp). **(H)** Ishak score.

To test the stability and reliability of the results of the meta-analysis, sensitivity analyses were performed on the outcome indicators, and it was found that excluding each study did not affect the magnitude of the combined effect, which indicates that this study has good stability and reliable results (Figure 11). (Supplementary Figure S4)

## 5 Discussion

Currently, a large number of anti-hepatic fibrosis drugs are available in the clinic, but their efficacy is not sufficient to reverse liver fibrosis, and liver transplantation is still a fast and effective way to treat liver fibrosis. However, due to the problems of donor scarcity and expensive operation costs, patients are often not treated in time. Recent studies have highlighted the potential of MSC-exos in reversing liver fibrosis, with their therapeutic value gaining public recognition. The smaller size, lower immunogenicity, and non-tumorigenicity of MSC-exos have positioned them as a promising strategy for treating hepatic fibrosis. Moreover, as researchers continue to explore this topic in greater depth, MSC-exos-drugs have also shown better efficacy in recent years. Compared with the treatment of MSC-exos single administration, the combination of MSC-exos and anti-hepatic fibrosis drugs further enhanced the degree of hepatic fibrosis reversal, which may offer valuable insights for future research and clinical application.

This paper includes a comprehensive analysis of 12 studies on the efficacy of MSC-exos in treating liver fibrosis through single administration, as well as six studies on the use of MSC-exos in combination with drugs. The comparison between the efficacy of MSC-exos-drugs and MSC-exos alone is also discussed. Our study focuses on the importance of monitoring various outcome metrics in the treatment of liver fibrosis. The reasons for choosing the corresponding outcome metrics are as follows: 1) the progression of various chronic liver diseases is usually due to an imbalance between the production and degradation of the extracellular matrix in the liver, which results in excessive collagen deposition, and the changes in collagen and fiber can be observed by using Sirius red staining and Masson staining (Parola and Pinzain, 2019; CalIgiuri et al., 2021). 2) The Ishak score and liver index can be used to evaluate the degree of hepatic fibrosis; in addition, α-SMA, as a marker of HSC activation, can also predict the progression of hepatic fibrosis (Beaussier et al., 2007). 3) ALT, AST, and ALP are mainly distributed in hepatocytes and are the most commonly used serum markers in the clinical diagnosis and treatment of patients with liver diseases. When the liver is damaged, ALT, AST, and ALP in hepatocytes enter the bloodstream, resulting in increase in their indicators (Woreta and Alqahtani, 2014; Kwo et al., 2017). Pooled analysis revealed that the degree of liver fibrosis improved with both MSC-exos single administration and MSC-exos-drugs. MSC-exos single administration in the treatment of liver fibrosis showed the following effects: ①reduced collagen deposition and fibroplasia (Masson and Sirius red staining); ②slowed down the progression of liver fibrosis (Ishak score, liver index, and α-SMA); ③improved liver function (AST, ALT, and ALP). For MSC-exos-drugs, the therapeutic effect is more significant compared with that of MSC-exos alone, which is mainly reflected in: ①reduction of collagen deposition (Masson staining); ②reduction of liver index; ③improvement of liver function (AST and ALT).

### 5.1 MSC-exos single administration for the treatment of liver fibrosis

In the field of exosome research, the sources, extraction methods, and infusion methods of exosomes vary, just as the parental cells of exosomes vary. Further subgroup analytic exploration is necessary to understand whether these differences lead to different efficacy. Similarly, there is some heterogeneity in the way liver fibrosis models are currently modeled and in the choice of species, which warrants further study. When considering MSC-exos for liver fibrosis treatment, it is crucial to examine the diverse outcomes influenced by these factors.

#### 5.1.1 Factors affecting MSC-exos

MSC-exos are widely used due to their advantages of small size, low immunogenicity, and non-tumorigenicity, among which there are many studies on hUC-MSC-exos and BM-MSC-exos. Recent studies suggest that hUC-MSC-exos may have a stronger therapeutic effect on osteoarthritis compared to BM-MSC-exos, hinting at the potential for targeted disease treatment using MSC-exos (Wang et al., 2022). The phenomenon has subsequently been confirmed by researchers in other countries. (Cai et al., 2020; Chen et al., 2020; Pomatto et al., 2021; Soni et al., 2021; Deng et al., 2022; Semerci et al., 2023). This may be caused by the variability of non-coding RNAs, proteins, and lipids in the contents of MSC-exos from different sources, but the low number of studies prevents this conclusion from being confirmed. In summary, it is important to consider the specific type of MSC-exos when selecting them for therapy.

In the included studies, the MSC-exos used were mainly from BM-MSCs, ADSCs, hUC-MSCs, and AMSCs. We performed MSC-exos source subgroup analyses of liver function, pathological histological changes, and the progression of hepatic fibrosis separately. AST and the Sirius red-stained area decreased in the hUC-MSC-exos subgroup, but there was no statistically significant difference in the other indicators; however, the α-SMA level decreased in the BM-MSC-exos subgroup, and there was no statistically significant difference in the other indicators. Moreover, all indicators decreased in the ADSC-exos subgroup. This finding shows that ADSC-exos demonstrated stability and superiority in liver fibrosis treatment, supporting the notion of targeted MSC-exos therapy. However, the existing research is not conclusive on which MSC-exos are the most effective in treating liver fibrosis, which is still an unresolved issue.

There are many ways to extract exosomes, and the ultracentrifugation method has been widely used as the “gold standard” for exosome extraction, but it also has certain limitations (e.g., equipment dependence, cumbersome operation, and time-consuming) (Colao et al., 2018; Kurian et al., 2021). Although the kit extraction method is fast and safe, with higher purity yield and a wide variety of products, its extraction results are uneven, has a high price, and is less cost-effective. Recent advancements in science and technology have introduced new separation techniques such as ultrafiltration, integrated dual-filtration microfluidic devices, nano-plasma enhanced scattering, membrane-mediated exosome separation, and exosome separation on a chip (Yu et al., 2018). Although the yield and purity of exosomes have improved to a certain extent with the improvement in new technology, they still face challenges such as high costs and equipment requirements. Additionally, combining extraction methods is gaining recognition. Some researchers have found that for the same source of sample for exosome separation, the combined extraction method is better than the individual extraction methods in terms of the overall efficiency of exosome purity and yield. Moreover, for different sample materials, the purity and yield of exosomes can be optimally balanced by combining different extraction methods (Stam et al., 2021). Different extraction methods can lead to differences in exosome levels, which may further affect the efficacy of the treatment of liver fibrosis. Therefore, when selecting MSC-exos for therapeutic purposes, careful consideration of the extraction method is essential.

Ultracentrifugation and kit extraction methods were the primary focus of the studies analyzed. We analyzed the subgroups of MSC-exos extraction methods for ALT and α-SMA, respectively. The results showed that ALT, AST, and α-SMA decreased regardless of the extraction method chosen. The forest plot of the subgroup analysis suggested that the kit extraction method showed a slight advantage in reducing ALT and AST levels, while ultracentrifugation was more effective in decreasing α-SMA. However, due to the limited number of studies included, further validation of this conclusion is necessary.

Common modes of exosome infusion include intravenous (tail vein-based, liver lobe injection, and penile vein injection), intraperitoneal, subcutaneous, and topical injections. Different modes of injection of MSCs affect the outcome of liver fibrosis (Chen et al., 2022), and a similar phenomenon may exist for exosomes and the substances they secrete. The distribution of exosomes in the organs of the body is affected by the different methods of injection. Exosomes are distributed mainly in the injection site or lymph nodes by subcutaneous injection, mainly in the gastrointestinal tract and lymph nodes by intraperitoneal injection, and mainly in the liver and spleen by intravenous injection (Patel et al., 2022). For effective treatment, it is crucial that exosomes are distributed in high quantities to the target organ. However, the optimal infusion modality for hepatic fibrosis treatment remains unknown and should be considered when using MSC-exos for disease management. Unfortunately, subgroup analyses of infusion patterns were not possible due to the small sample size.

In addition to variations in the source, extraction method, and infusion technique of MSC-exos, some scholars have found, in recent years, that the differences in the cultivation method and the environment of the parental cells of MSC-exos may also affect the yield, purity, or the content of the internal substances in the extraction of MSC-exos, which may ultimately lead to the differences in the therapeutic efficacy of hepatic fibrosis.

Haraszti observed that MSCs cultured in a 3D environment produced 20 times more MSC-exos compared to a 2D culture when isolated using conventional ultracentrifugation, highlighting the potential of 3D culture for enhanced MSC-exos isolation, which is crucial for both research and clinical applications (Haraszti et al., 2018; Zhang, 2023). It was also found that long-term hypoxic culture of MSCs did not affect the content of soluble cytokines secreted by the cells (e.g., IL-10 and TGF-β), but rather the expression of miRNAs in hUC-MSC-exos (Wu, 2022). Similarly, Wang Qi et al. reported that the differences between culture conditions affect the changes in the internal protein content of MSC-exos in addition to the yield of MSC-exos (Wang, 2022). In addition to the above influences, the environment in which the cells are exposed, including oxidative stress, inflammation, and the tumor microenvironment, also affects the extraction of MSC-exos (Jafari et al., 2020). In conclusion, when utilizing MSC-exos for liver fibrosis treatment, it is essential to consider the source, extraction method, infusion technique, parental cell environment, and the differences in exosomes resulting from the extraction strategy.

#### 5.1.2 Factors affecting animals with liver fibrosis

In addition to considering the influence of MSC-exos, differences in efficacy due to heterogeneity in the types of animals with liver fibrosis and the modeling modality are worth exploring.

There is a wide variety of species sources for liver fibrosis models. As animals and humans differ on many levels, the conclusions drawn from animal models can only be used as a reference material for clinical applications. It is important to select animals that are biologically closer to humans to ensure that the animal model can truly reflect the characteristics and course of human liver fibrosis and make the conclusions more informative. Therefore, we need to consider which type of animal to choose when conducting animal experiments.

The included studies mainly involved mice vs rats. We analyzed the experimental animal subgroups for liver function, pathological histological changes, and hepatic fibrosis progression. The results showed that ALT, AST, Masson-stained area, and α-SMA decreased in the mice subgroup, while the rat subgroup showed no statistical significance, except for a decrease in the Masson-stained area. It has been shown that rats are more similar to humans biologically and physiologically and are more suitable as an animal model for liver fibrosis (Yang et al., 2018; George et al., 2020). However, it is clear from the above subgroup analysis that MSC-exos is unstable for the treatment of liver fibrosis in rats. Put another way, however, in terms of the given metrics of improvement in liver fibrosis, without regard to the presence or absence of statistical significance, MSC-exos are more effective in treating liver fibrosis in rats than in mice (Figure 6 and Supplementary Figures S2 B, S3 B), which has similarities to the findings of others mentioned above, although the disadvantage of instability is still evident. To investigate the reasons for the instability of MSC-exos for the treatment of liver fibrosis in rats, we further explored the types of MSC-exos used for the treatment of liver fibrosis in rats and mice. Surprisingly, it was found that in the included studies, BM-MSC-exos was mainly used for the treatment of liver fibrosis in rats, while hUC-MSC-exos, ADSC-exos, and TMSC-exos were mainly used for the treatment of mice. This phenomenon reminds us once again that, on the one hand, we need to consider the existence of differences in the efficacy of different sources of MSC-exos in the treatment of hepatic fibrosis, and on the other hand, when treating liver fibrosis in rats and mice or other animals, we also need to consider the source of MSC-exos. From the results of the subgroup analyses above, we can draw a conclusion that for the treatment of liver fibrosis, there may be poor stability in the use of BM-MSC-exos. In order to obtain more accurate conclusions, a large number of experiments are needed to validate the stability of MSC-exos in the treatment of hepatic fibrosis in rats and validate the issue of the superiority of rats as a model of hepatic fibrosis.

Currently, *in vivo* models of liver fibrosis can be categorized into five groups based on the etiology: chemical, dietary, surgical, transgenic, and immunological. The variety of animal models of liver fibrosis and their varying efficacy have different implications for the proper understanding of the disease and the effective screening of therapeutic agents (Wu et al., 2023). The primary focus of the studies reviewed in this paper was on CCl4, TAA, and DEN as inducers of liver fibrosis. Among these, the CCl4-induced liver fibrosis model shares similarities with human liver fibrosis in certain morphological and pathophysiological aspects, making it the most commonly utilized method due to its efficiency, cost-effectiveness, and reproducibility. Considering that differences in the modeling modality of liver fibrosis may also affect the efficacy of MSC-exos, this subgroup analysis needs to be considered; unfortunately, the requirement for subgroup analysis of modeling modalities could not be met due to the small sample size in the current analysis.

In summary, from the results of the subgroup analysis of outcome indicators such as Masson-stained area, α-SMA, AST, and ALT, it can be seen that, first, regardless of which extraction method was chosen, MSC-exos were efficacious at improving hepatic fibrosis, and there was only variability in the effects of different indicators. Second, the efficacy of BM-MSC-exos and hUC-MSC-exos in the treatment of hepatic fibrosis was mixed, while the efficacy of ADSC-exos was generally stable. Finally, there was also uncertainty in the efficacy of MSC-exos in the treatment of rats as a model of liver fibrosis, whereas the mice subgroup had better efficacy. Therefore, when treating liver fibrosis, it is advisable to consider using ADSC-exos, which supports the potential targeting of MSC-exos for this disease. In addition, it is important to consider the impact of the modeling species and the MSC-exos extraction method on the experiment. However, due to the limited number of included articles, several studies need to be performed to validate these findings.

### 5.2 MSC-exos-drugs for liver fibrosis

Recent research has focused on the potential of MSC-exos-drugs to improve the efficacy of liver fibrosis treatment. Therefore, a meta-analysis of MSC-exos-drugs was conducted.

As shown by the pooled analysis, Masson-stained area, AST, and ALT level were significantly lower in the MSC-exos-drug group than in the MSC-exos single administration group. We performed subgroup analyses of serum liver function and pathological tissue changes for the MSC-exos injection method, extraction method, and model species, respectively. The results showed that each index decreased in the intraperitoneal injection subgroup, the mice subgroup, and the kit extraction subgroup, while no statistical significance was found in the tail vein injection subgroup, the rat subgroup, and the ultracentrifugation subgroup, except for a slight decrease in the ALT index. From the above results, for MSC-exos-drugs, a more stable end result was obtained by kit extraction. Similarly, infusion through the abdominal cavity is more effective. However, the limited number of studies and variations in drug concentration, administration route, and treatment frequency suggest that more research is needed to confirm these findings. Overall, MSC-exos-drugs appear to be more effective than MSC-exos single administration in treating liver fibrosis.

In addition to the effects of MSC-exos-drugs, the anti-hepatic fibrosis capacity of MSC-exos can be further enhanced through various methods, such as ① MSC-exos combined genetic engineering: MSC-exos modified with HSTP1 or combined with vitamin A can enhance the ability to target aHSCs and improve the efficacy of hepatic fibrosis by inhibiting HSC activation (You et al., 2021; Lin et al., 2022; Zhang YW. et al., 2023). ② MSC-exos combined drug loading technology: MSC-exos improves its drug-loading efficiency using gentle ultrasound, which in turn improves liver function (Kim et al., 2016; Ashour et al., 2022; Azizsoltani et al., 2023). ③ MSC-exos combined with different extraction methods: the extraction method of MSC-exos (alone or in combination), the culture method of MSCs, the environment, and the source of MSCs can affect the yield and purity of MSC-exos. (Haraszti et al., 2018; Jafari et al., 2020; Stam et al., 2021; Chen et al., 2022; Patel et al., 2022; Wang, 2022; Wang et al., 2022; Wu, 2022; Zhao et al., 2022; Zhang, 2023). It might be possible to choose the best protocol based on the factors affecting the extraction of MSC-exos to achieve the optimal balance between exosome yield and purity and, thus, improve its ability to fight liver fibrosis. ④ MSC-exos combined gene modification: MSC-exos further ameliorates liver fibrosis by overexpression of miRNAs or circRNAs (Qu et al., 2017; Ma et al., 2023). ⑤ MSC-exos in combination with other modalities: overexpression of HGF and MSC-exos preconditioning, among others, further delayed liver fibrosis progression (Takeuchi et al., 2021; Yu et al., 2023). Although MSC-exos-drugs have shown significant benefits, the modalities themselves are not yet well-researched as to whether they are harmful to cells, tissues, or the organism.

### 5.3 Limitations in this Meta-analysis

There are several limitations in this meta-analysis. 1) The number of studies of MSC-exos-drugs is insufficient, leading to the conclusion drawn above being still debatable. 2) Among the outcome indicators related to liver fibrosis treatment, the limited number of included studies did not allow for the extraction of sufficient indicators of inflammation to allow pooled and subgroup analyses to be performed. 3) As can be seen from the forest plot, much of the interstudy heterogeneity was high, and although subgroup analyses and sensitivity analyses were performed, the exact source of the heterogeneity was still unclear, and subgroup analyses could not be performed due to the high variability in treatment frequency, injection dose, and MSC-exos size between studies, and the heterogeneity was considered to exist for the above reasons. 4) Insufficient data due to the limited number of studies prevented the detection of publication bias.

## 6 Conclusion

In conclusion, in preclinical liver fibrosis models, both MSC-exos single administration and MSC-exos-drugs can reduce collagen deposition, fibroplasia, and improve liver function and, thus, reverse liver fibrosis. Moreover, MSC-exos-drugs exhibit additional benefits in reducing AST, ALT levels, and Masson-stained areas. Before the use of MSC-exos in clinical studies, there is an urgent need to establish a standard regimen for the treatment of hepatic fibrosis to fully utilize the potential of MSC-exos, which mainly involves the route of injection, frequency of injection, and injection dose. It is also important to consider the source of the MSC-exos, the method of extraction, and the combination therapy, which may further enhance the therapeutic effect.

## Data Availability

The original contributions presented in the study are included in the article/Supplementary Material; further inquiries can be directed to the corresponding author.

## Supplementary material

SUPPLEMENTARY MATERIAL S1
**(A)**: Subgroups of the MSC-exos source for α-SMA level. **(B)**: Subgroups of experimental animals for α-SMA levels. **(C)**: Subgroups of MSC-exos extraction methods for α-SMA levels.

SUPPLEMENTARY FIGURE S2)
**(A)**: Subgroup of the MSC-exos source for AST level.

SUPPLEMENTARY FIGURE S3
**(A)**: Subgroups of the MSC-exos extraction method for ALT level. **(B)**: Subgroups of experimental animals for ALT levels.

SUPPLEMENTARY FIGURE S4 Sensitivity analysis (MSC-exos combination drug therapy). **(A)** Alanine aminotransferase (ALT). **(B)** Aspartate aminotransferase (AST). **(C)** Liver index. **(D)** Masson-stained area.

SUPPLEMENTARY MATERIAL S2 Detailed search strategy.

## Abbreviations

MSCs, mesenchymal stem cells; MSC-exos, mesenchymal stem cell exosomes; BM-MSCs, bone marrow-derived mesenchymal stem cells; BM-MSC-exos, bone marrow-derived mesenchymal stem cell exosomes; hUC-MSCs, human umbilical cord-derived mesenchymal stem cells; hUC-MSC-exos, human umbilical cord-derived mesenchymal stem cell exosomes; ADSCs, adipose-derived mesenchymal stem cells; ADSC-exosomes, adipose-derived mesenchymal stem cell exosomes; TMSCs, tonsil mesenchymal stem cells; TMSCs-exos, tonsil mesenchymal stem cell exosomes; AMSCs, amniotic mesenchymal stem cells; AMSCs-exos, amniotic mesenchymal stem cells exosomes; ALB, albumin; ALT, alanine aminotransferase; AST, aspartate aminotransferase; ALP, alkaline phosphatase; Hyp, hydroxyproline; α-SMA, α-smooth muscle actin; HSCs, hepatic stellate cells; aHSCs, activated hepatic stellate cells; TGF-β, transforming growth factor-β; COL-I, type I collagen; COL-III, type III collagen; HGF, hepatocyte growth factor; TNF-α, tumor necrosis factor α; MSC-exos-drugs, MSC-exos in combination with drugs; CCl_4_, carbon tetrachloride; TAA, thioacetamide; DEN, diethylnitrosamine; SMD, standardized mean difference; TIMPs, tissue inhibitor of metalloproteinases.
