# Supplementary material for: Single and combined strategies for mesenchymal stem cell exosomes alleviate liver fibrosis: a systematic review and meta-analysis of preclinical animal models
Source: Front Pharmacol. 2024 Jul 31;15:1432683. doi: 10.3389/fphar.2024.1432683 (PMC11322148; doi:10.3389/fphar.2024.1432683)
Supplement: Supplementary file 2 [file DataSheet1.docx]

**Supplementary Materials**

S1

A


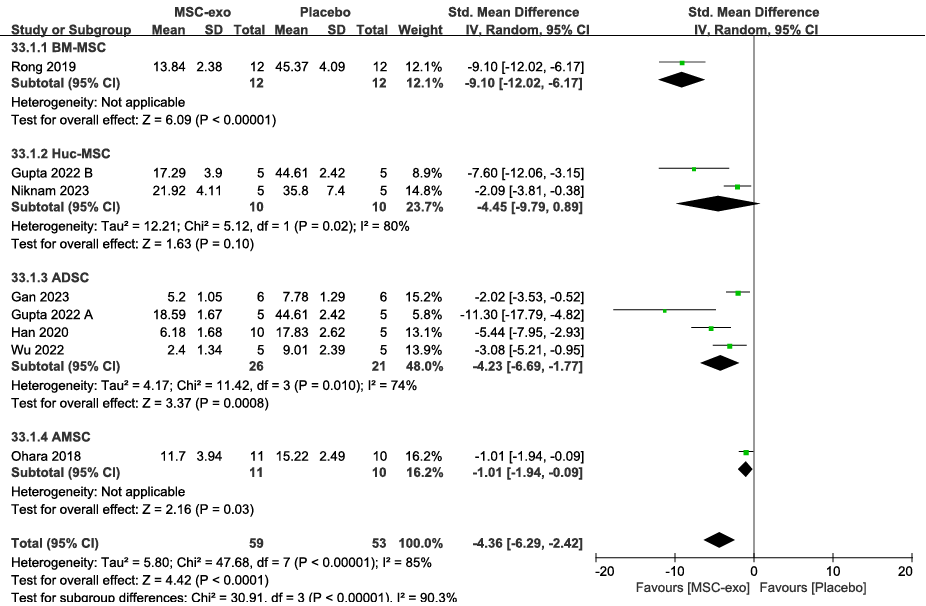


B


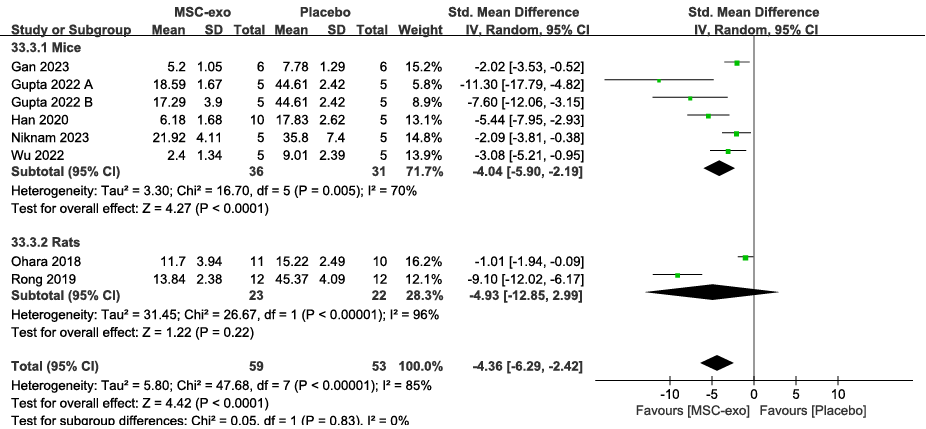


C


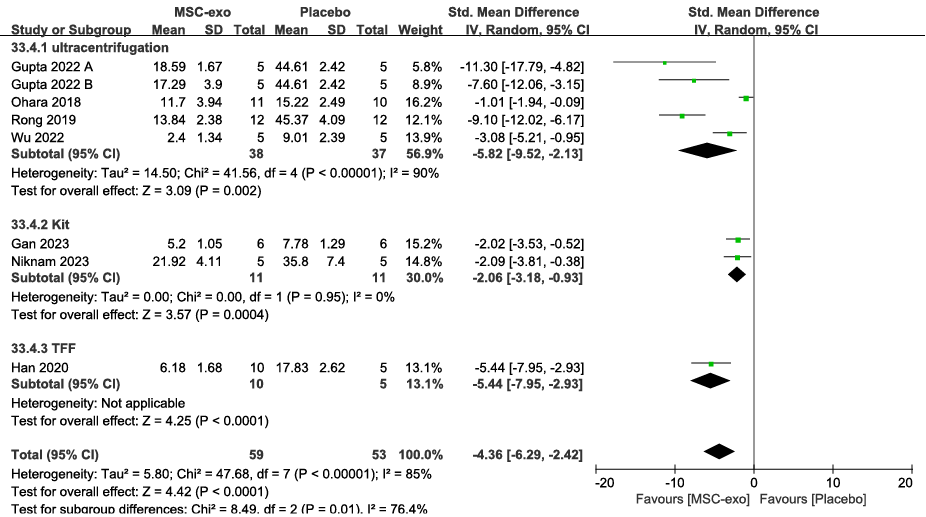


**Figure S1** A: Subgroups of MSC-exos source for α-SMA level; B: Subgroups of experimental animals for α-SMA level; C:Subgroups of MSC-exos extraction methods for α-SMA level

S2

A


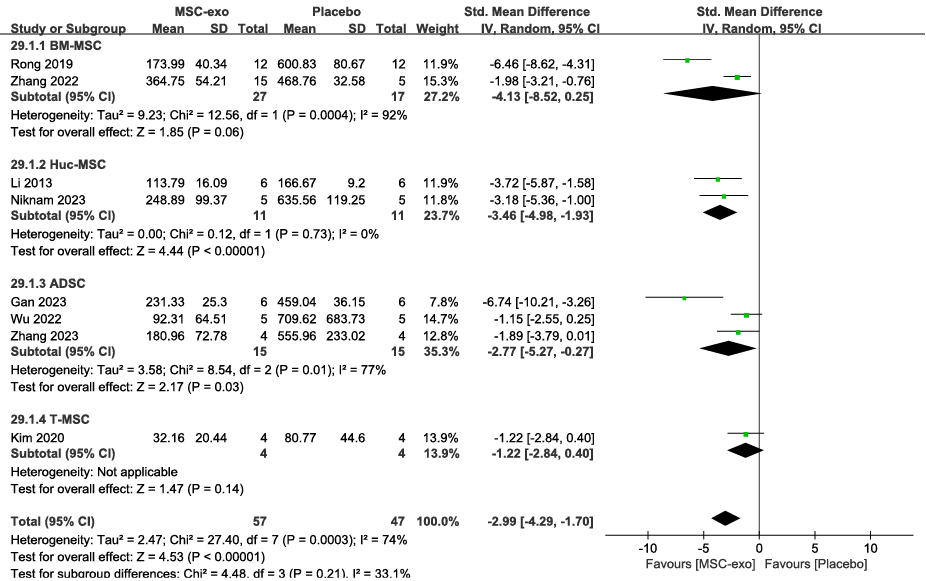


**Figure S2**  Subgroups of MSC-exos source for AST level

**S3**

**A**


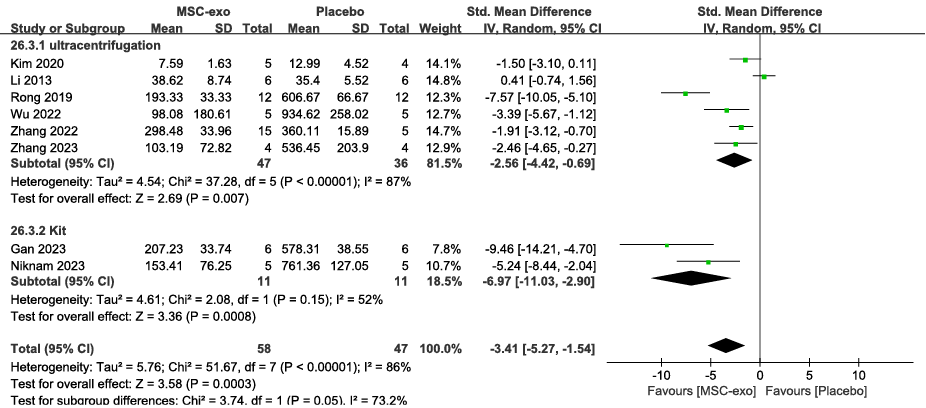


**B**


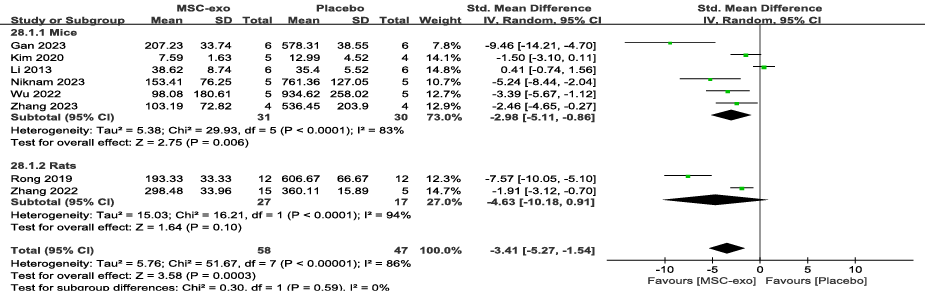


**Figure S3** A: Subgroups of MSC-exos source for ALT level; B: Subgroups of experimental animals for ALT level

**S4**


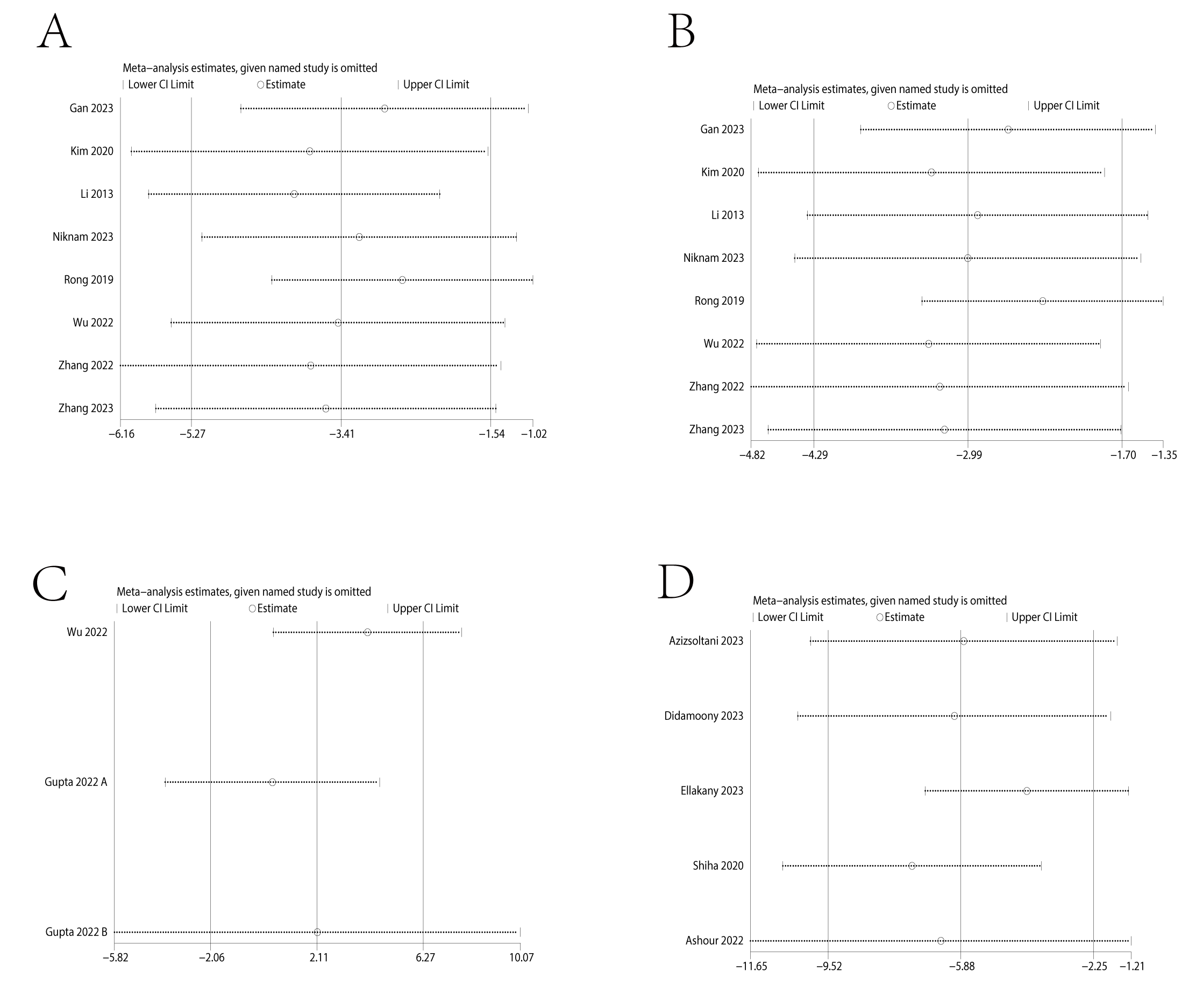


**Fig. S4** Sensitivity analysis (MSC-exos combination drug therapy). **A** Alanine aminotransferase(ALT). B Aspartate aminotransferase(AST). **C** Liver index. **D** Masson staining area.
